# A systematic review to examine the evidence regarding discussions by midwives, with women, around their options for where to give birth

**DOI:** 10.1186/s12884-016-0832-0

**Published:** 2016-03-14

**Authors:** Catherine Henshall, Beck Taylor, Sara Kenyon

**Affiliations:** Public Health, Epidemiology and Biostatistics, Institute of Applied Health Research, University of Birmingham, Birmingham, B15 2TT UK

**Keywords:** Place of birth, Midwives, Systematic review

## Abstract

**Background:**

Discussion of place of birth is important for women and maternity services, yet the detail, content and delivery of these discussions are unclear. The Birthplace Study found that for low risk, multiparous women, there was no significant difference in neonatal safety outcomes between women giving birth in obstetric units, midwifery-led units, or home. For low risk, nulliparous women giving birth in a midwifery-led unit was as safe as in hospital, whilst birth at home was associated with a small, increased risk of adverse perinatal outcomes. Intervention rates were reduced in all settings outside hospital. NICE guidelines recommend all women are supported in their choice of birth setting.

Midwives have the opportunity to provide information to women about where they choose to give birth. However, research suggests women are sometimes unaware of all the options available.

This systematic review will establish what is known about midwives’ perspectives of discussions with women about their options for where to give birth and whether any interventions have been implemented to support these discussions.

**Methods:**

The systematic review was PROSPERO registered (registration number: CRD42015017334). The PRISMA statement was followed. Medline, Cochrane, CINAHL, PsycINFO, Popline and EMBASE databases were searched between 2000-March 2015 and grey literature was searched. All identified studies were screened for inclusion. Qualitative data was thematically analysed, whilst quantitative data was summarised.

**Results:**

The themes identified relating to influences on midwives’ place of birth discussions with women were organisational pressures and professional norms, inadequate knowledge and confidence of midwives, variation in what midwives told women and the influence of colleagues. None of the interventions identified provided sufficient evidence of effectiveness and were of poor quality.

**Conclusions:**

The review has suggested the need for a pragmatic, understandable place of birth dialogue containing standard content to ensure midwives provide low risk women with adequate information about their place of birth options and the need to improve midwives knowledge about place of birth. A more robust, systematic evaluation of any interventions designed is required to improve the quality of place of birth discussions. By engaging with co-produced research, more effective interventions can be designed, implemented and sustained.

## Background

Discussion of place of birth (PoB) is important for women and maternity services, yet presently the detail, content and delivery of these discussions are not clear. We have little understanding of midwives’ perceptions of PoB discussions with women, including what ‘good’ PoB discussion should look like, or what challenges midwives face in integrating PoB discussion into their practice. The Birthplace Study [1] found that for low risk, multiparous women, there was no significant difference in neonatal safety outcomes between women who gave birth in obstetric units (OUs), midwifery-led units (MLUs), or at home. It also found fewer maternal interventions in women giving birth at home and a low transfer rate of 12 %. The study also found that for low risk, nulliparous women, giving birth in a MLU is just as safe as giving birth in hospital, although there is a small but increased chance of adverse perinatal outcomes, which include intrapartum stillbirth, early neonatal death, neonatal encephalopathy, meconium aspiration syndrome, and specified birth related injuries including brachial plexus injury, if the baby is born at home. Nulliparous women are more likely to have a normal birth with fewer complications and better recovery at home or in an MLU and are less likely to have a caesarean section [1]. Offering choice in PoB may also increase satisfaction, with many studies reporting increased satisfaction with non-OU settings [2, 3]. Many National Health Service (NHS) maternity trusts have seen increases in the birth rate in recent years and increasing the uptake of other birth settings may ease the pressure on inpatient service capacity. Recently published National Institute for Health and Care Excellence (NICE) guidelines have also recommended that both multiparous and nulliparous women should be supported in their choice of birth setting, wherever they choose to give birth [4].

Midwives have the opportunity to provide information and discussion to women about where they choose to give birth. This usually occurs at the first antenatal booking appointment. However, research suggests women are sometimes unaware of the range of PoB options and that many may be interested in a birth outside of an OU if it is discussed [5, 6].

This systematic review will examine the existing literature regarding discussions by midwives, with women, around their options for where to give birth. It will establish what is known about midwives’ perspectives of discussions with women about their options for where to give birth. In addition the systematic review will explore whether any interventions have been implemented to support midwives’ PoB discussions with women. For all interventions identified, the review will examine their effectiveness and any barriers and facilitators to implementation.

Thus, this systematic review will aim to answer the following questions:

- **Review 1**: What is known about midwives views of their discussions with women about their options for where to give birth?
- **Review 2**: Have any interventions been implemented to support midwives’ PoB discussions with women? If so, what were the barriers and facilitators to implementing them and have the interventions been effective?

## Methods

The systematic review was registered by PROSPERO (registration number CRD42015017334) [7]. The PRISMA statement was followed [8, 9].

### Criteria for considering studies for this review

#### Types of studies

All systematic reviews, randomised or quasi- randomised controlled trials, observational studies or qualitative studies were considered for inclusion if they focused around PoB discussions between midwives and women, from midwives perspectives. Studies were excluded from the review if they focused around place of birth discussions between midwives and women, from the perspective of the women (or anyone else except the midwife), if they were published before the year 2000, or if they were carried out outside of Europe, North America or Australasia.

#### Types of participants

The population was limited to midwives working in any midwifery setting, who were practising or had practised in Europe, North America or Australasia.

#### Types of Interventions (review 2 only)

The review included studies that explored the effectiveness, barriers or facilitators, of an intervention aimed at supporting midwives PoB discussions with women. Midwives must have received, or been responsible for delivering the intervention.

#### Search methods for identification of studies

The databases Medline, Cochrane Database, CINAHL, PsycINFO, Popline and EMBASE were searched between February-March 2015. Both text and indexed terms (such as MeSH) were used and modified as necessary in each database. An example of the search strategy used is given in Table 1.

**Table 1:** Example of Search Strategy from Medline (R) 1946 to February Week 1 2015

	Searches
1	(birthplace or place of birth) ti,ab.
2	((home or hospital or institut$ or place or locat$ or setting$) adj3 (birth$ or confine or confinement or confining or deliver$)). ti,ab.
3	Home childbirth.mp. or Home Childbirth/
4	Delivery, Obstetric/px
5	1 or 2 or 3 or 4
6	(choice or preference or decision$ or dialog$ or discussion$ or consultation$ or conversation$ or communication$ or attitude$ or perspective$ or view$). Ti,ab. [mp = title, abstract, original title, name of substance word, subject heading word, keyword heading word, protocol supplementary concept word, unique identifier]
7	Patient Preference/
8	(Women$ adj3 Preference$).ti,ab
9	Midwifery/
10	(midwife$ or midwives)ti,ab. [mp = title, abstract, original title, name of substance word, subject heading word, keyword heading word, protocol supplementary concept word, unique identifier]
11	6 and 10
12	7 or 8 or 9 or 11
13	5 and 12

Grey literature was also searched, through the Department of Health, Royal College of Midwives (RCM), Royal College of Obstetricians and Gynaecologists, National Childbirth Trust, Association for Improvements in the Maternity Services, Maternity Action, Which? Birth Choice, NHS England and King’s Fund, websites. Google and Google Scholar were searched for any relevant, unpublished studies. Reference lists of key full text articles included in the review were checked to identify any potentially eligible studies.

Searches were limited to papers in the English language, published between 2000-2015, due to changes in midwifery practice making it unlikely that relevant articles would be found before this time.

#### Selection of studies

All identified studies were screened for inclusion in the review, based on the study eligibility criteria. To be included in the review, identified studies had to meet either the eligibility criteria outlined above, for either review one, review two, or both.

#### Data extraction and risk of bias

Data from the included studies was extracted independently, discussed and summarised, by two reviewers (CS, BT) using a data extraction form which was constructed to collect all relevant study data. This data is summarised in Tables 2 and 3. The quality of the studies was also assessed by the same reviewers (CS, BT), using the relevant Critical Appraisal Skills Programme checklists [10]. Any disagreement about the criteria or level of bias was discussed until a mutual decision was reached. Where necessary the study authors were contacted to obtain more detailed information.

**Table 2:** Summary characteristics of studies (Review 1)

Study	Study country	Midwifery setting	Study aim	Study design	Analysis methods	Number of participating midwives	Total Risk of Bias within study
Barber et al (phase 1). 2006 [15]	UK	Two NHS Trusts, each with obstetric units, alongside and free-standing maternity led units and homebirth services.	To identify factors that influence women’s decisions about where to give birth.	Qualitative. Focus groups with midwives.	Thematic analysis.	16	High
Davis et al. 2010 [16]	New Zealand	Case-loading midwives, so move between home to hospital.	To explore the way case-loading midwives construct midwifery and to examine their practice within the obstetric hospital	Qualitative, in-depth interviews.	Thematic analysis.	48	High
				Feminist, post-structuralist framework.			
Lavender et al. 2004 [17]	UK	14 sites, comprising home birth settings, free-standing midwifery-led units, alongside midwifery-led units and obstetric units.	To explore the views of midwives working in maternity services, in relation to birth setting, models of care and philosophy of care.	Qualitative. Focus groups with midwives.	Thematic analysis.	126	Low
				Appreciative inquiry.			
RCM, 2011 [18]	UK (97 %, *n* = 536) and outside of UK (3 %, *n* = 17)	Community, integrated community and hospital setting, midwifery led units, hospital obstetric unit and other settings.	To gain a national picture of midwives’ current thinking about home birth practice and to identify areas of concern by midwives and any education and practice needs in this area.	Quantitative. Online survey.	Descriptive statistics.	553	High
Vedam et al. 2009 [19]	North America	Urban centres, rural areas and a mixture of both settings.	To describe the attitudes and experiences of midwives toward planned home birth and to explore evidence-based correlates and predictors of their attitudes toward planned home birth.	Quantitative. Online and paper survey.	Descriptive statistics; correlation analysis.	1893	High
Vedam et al. 2012 [20]	Canada	Registered midwives working in any setting.	To describe educational, practice and personal experiences related to home birth among obstetricians, family physicians, and registered midwives; to identify barriers to provision of planned home birth services and examine inter-professional differences in attitudes towards planned home birth.	Quantitative. Online survey.	Descriptive statistics; correlation analysis.	451	High

**Table 3:** Summary characteristics of studies (Review 2)

Study	Study country	Midwifery setting	Study aim	Study design	Analysis methods	Number of participating midwives	Total Risk of Bias within study
Barber et al (phase 2). 2006 [12]	UK	Two NHS Trusts, each with obstetric units, alongside and free-standing maternity led units and home birth services.	To implement educational, marketing and change management initiatives on and around informed choice and place of birth. This included relaunching the Birth Centres at both Trusts, the provision of local evidence-based leaflets with information on all the birth place options and Birthplace Choices websites for each Trust.	Qualitative Interactive, educational interventions with midwives.	Qualitative feedback from intervention session.	38 participated in workshops.	High
Barber et al (phase 3). 2007 [22]	UK	Two NHS Trusts, each with obstetric units, alongside and free-standing maternity led units and home birth services.	To evaluate which initiatives helped midwives promote informed choice around place of birth. To identify if more women had subsequently chosen an out of hospital birth.	Quantitative survey study with midwives.	Descriptive statistics.	150	High
Kirkham et al. 2001 [21]	UK	Three maternity units, encompassing community, hospital, integrated hospital and community case-loading and specialist roles.	To assess the impact of the MIDIRS Informed Choice Leaflets (for health professionals) on health professionals.	Qualitative ethnographic and interview study.	Ethnographic field notes and grounded theory approach to interview analysis.	177	Unclear
Rogers et al. 2015 [13]	UK	One large hospital maternity unit	To improve informed choice and the knowledge and confidence of midwives around place of birth using workshops for women and midwives, ‘decision aid’ tools and changes to the midwifery rota.	Mixed methods: Workshops and survey study.	Qualitative feedback from workshops.	Not reported.	High
					Descriptive statistics to summarise survey data.		
Walton et al. 2014 [11]	UK	One large hospital maternity unit	To increase the number of women having a clear preference for place of birth, ideally by 36 weeks, using the Birthplace app intervention (introduced at 25 weeks).	Quantitative. Pilot controlled study.	Descriptive statistics. Retrospective analysis of data collected at booking visit, 12 and 36 weeks on women’s choice of place of birth setting.	35	High

#### Missing data

Authors of three included studies were contacted to request data on the number of midwives who had participated in the studies. Two study authors provided this data [11, 12], whilst no response was received from the third [13]. Therefore this data is missing from the review findings.

#### Data synthesis

The qualitative data in the review was thematically analysed [14], allowing us to establish any commonalities in midwives’ discussions with women, around their options of where to give birth, within and between the studies. Quantitative data from the included studies was summarised. However, due to the variety of study designs included in the review and the poor quality of the statistical data (which was largely descriptive) meta-analyses of the data was not appropriate, as a pooling of results across studies was not possible.

## Results

### Characteristics of included studies

**Fig. 1:**
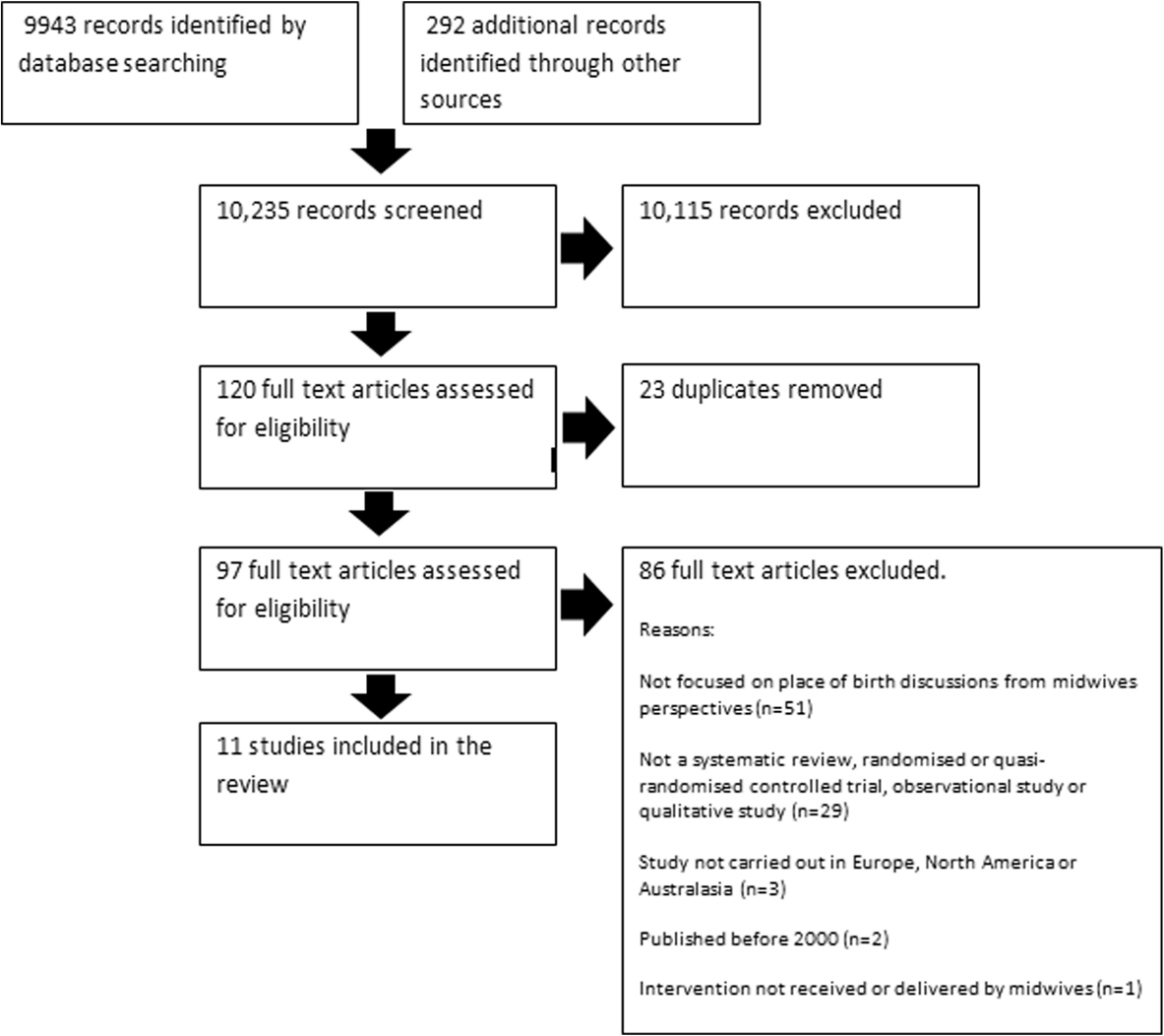
Flow diagram to show the number of articles screened for inclusion in the systematic reviewᅟ

10,235 records titles and abstracts were initially screened for inclusion in the review, of which 10,115 were excluded, leaving 97 articles remaining, following the removal of any duplicates (*n* = 23). Of these, 86 were excluded once the full article was assessed for eligibility, leaving 11 articles for inclusion in the review overall (Fig. 1).

#### Review 1: Midwives views of the discussions with women about their options for where to give birth

Characteristics of the six studies which met the inclusion criteria are shown in Table 2 [15–20]. All studies were multi-sited and took place in a range of urban and rural midwifery settings including consultant led OUs, MLUs and homebirth settings. Midwives either provided case-loading care, were hospital or community based, or worked in both settings. Three of the studies took place in the United Kingdom (UK) [15, 17, 18] (with one also having a small number of participants from outside of the UK) [18], one in the United States (US) [19], one in Canada [20] and one in New Zealand [16] and they were published between 2004 and 2012. In total 3087 midwives were included, with the number of midwives in the studies ranging from 16–1893. Three of the studies had qualitative [15–17] and three quantitative [18–20] designs. The studies were assessed using CASP tools and five were found to have a high risk of bias [15, 16, 18–20] and one a low risk of bias [17]. Though the studies methodologies were generally robust and used appropriate study designs for answering the research questions, many studies lacked precision and detail when reporting the study findings and showed lack of rigour in their data analysis techniques. For example some studies provided little or no details of the data analysis methods and techniques they used, showed no justification for the study sample size, failed to report any significant associations or failed to describe any potential confounding factors.

**Table 4:** Summary of findings (Review 1)

Resource issues	Organisational and professional norms	Influence of midwifery colleagues	Midwives’ perspectives on their role in women’s decision-making	Confidence of midwives	Strategies for improvement
• Lack of midwifery staff • Time pressures • Resource implications (unit closures)	• Hospital policies/organisational pressures • Peer opinion • Pressure to conform to status quo • Concerns about litigation	• Conflicting opinions about place of birth settings • Lack of homebirth promotion • Unsupportive attitudes towards homebirth	• Importance of offering range of choices • Booking visit wrong time for discussion • Women’s decision-making unaffected by discussion • Cultural/societal factors, and parity, influence women’s decision-making • Women’s responsibility to explore options • Importance of revisiting options with women	• Varying levels of confidence around discussing homebirth • Unwillingness and uncertainty of offering homebirth • Lack of skill and confidence in different birth settings	• Training in discussion of risk and promotion of homebirth • Leaflet focusing on birthplace choices for women

#### Qualitative studies

Three studies used qualitative methods. The first focus group study by Barber et al [15] aimed to identify factors influencing women’s decisions about where to give birth. The second focus group study by Lavender et al [17] aimed to explore midwives’ views about birth setting, models and philosophies of care. The third, qualitative study, by Davis et al [16], consisted of in-depth interviews with midwives, about how they constructed midwifery and examined their practice in obstetric settings.

#### Quantitative studies

The three quantitative studies included involved online surveys with midwives, with two also using paper-based formats [19, 20]. The RCM [18] study aimed to gain a national picture of midwives thinking about the adequacy of information given to women about homebirth. Vedam et al [19] collected data on the attitudes and experiences of midwives toward planned homebirth. Vedam et al [20] collected data on the educational, practice and personal experiences related to homebirth practice, barriers to the provision of planned homebirth services, and inter-professional differences in attitudes towards planned homebirth.

#### Study findings

Thematic analysis of the papers identified demonstrated a number of common themes, which are detailed below. Data from the individual studies is presented in Tables 4 and 5 (a - b). The main findings that influenced midwives place of birth discussions with women related to midwives’ organisational and professional norms, their knowledge and confidence around discussing birth place options with women, differences in information exchanges with women and the influence of midwifery colleagues (table 4). The main findings regarding the examination of any interventions to support midwives PoB discussions with women related to the effectiveness of the intervention and any influences on intervention implementation (Table 5).

**Table 5:** Summary of findings (Review 2)

Effectiveness of intervention	Barriers to implementation
• Kirkham et al. (2001) [21] No evidence that leaflets effective in increasing women’s informed choice. Non-significant reduction in planned hospital birth. • Rogers et al. (2015) [13] Women who attended workshops and received decision aid tool more likely to be offered a choice of place of birth and receive sufficient information. Admissions to alongside midwifery led units increased, admissions to free-standing midwifery led units decreased and home births remained constant. • Walton et al. (2014) [11] More women decided a preferred place of birth setting following introduction of app. Absence of comparator group. Midwives found app useful communication tool. • Barber et al. (2007): No results reported on intervention effectiveness. Midwives increasingly engaged and tried to disseminate more leaflets to women. Most midwives found leaflet and multi-professional guideline useful, and a few subsequently changed their practice. Increase in women choosing out-of-hospital births reported in one Trust, a decrease reported in the other.	• Midwives’ personal experiences, views and philosophies influenced type of information and support given. • Limited information provided, depending on assessment of women’s risk • Difficulties providing sustainable home birth service. • Little value placed on leaflets as vehicles for change • Maintaining the status quo • Inappropriate use of leaflets

#### Organisational and professional norms

Overall, seven studies identified organisational and professional norms as influential in determining how midwives discussed PoB options with women [12, 15–17, 19–21]. Five studies described how midwives felt pressured to recommend obstetric led birth settings or be selective in the PoB options they presented to women, due to hospital policies, organisational pressures [16, 19, 21] and peer opinion [19], with some midwives wanting to avoid confrontation with their medical colleagues [15, 21]. Lavender et al [17] reported that midwives felt that their personal philosophy of care could be altered by working in certain birth settings, due to pressure to conform. Litigation concerns were reported to sometimes lead midwives to steer women towards making decisions about where to give birth which reaffirmed the status quo of their organisation [21].

Three studies [12, 15, 20] cited a lack of midwifery staff as a perceived barrier to midwives offering homebirth (or birth in alternative settings) to women. This was due to two midwives being required at a homebirth, leaving the maternity services understaffed and impacting on midwives’ ability to provide choice to women due to concerns about equity of care. Midwives were also observed to work under considerable time pressures, limiting their opportunities for discussion with women. This was viewed as a reason why only select information was passed onto women during PoB discussions [21] and could result in midwives making assumptions about women’s information needs.

Some midwives reported how they refrained from offering women the full range of birth place options [17], due to the closure of some MLUs and resulting lack of available choice.

#### Knowledge and confidence of midwives

In five studies, differences were demonstrated in midwives’ knowledge and confidence around discussing birth place options with women, particularly regarding homebirth. Midwives in the study by Rogers et al [13] had been unaware of many of the Birthplace study findings prior to the implementation of the intervention. Some midwives described feeling very confident promoting homebirth to women as a normal option [16, 18], with Vedam et al [20] reporting that few midwives felt uncomfortable discussing homebirth with women, believing it to be as safe as hospital birth. This contrasts with Vedam et al [19], who indicated that 41 % (*n* = 776) of midwives would not consider offering the option of homebirth to their own women, whilst another 14 % (*n* = 265) were unsure or unmotivated to do so. In Kirkham et al [21], midwives reported how lack of skill and confidence of different birth settings influenced the scope of PoB settings presented to women.

#### Information exchanges between midwives and women

Four studies reported on some of the factors influencing midwives’ PoB discussions with women. Midwives in the study by Barber et al [15] reported that they generally provided verbal information on PoB choices at women’s booking visits [22], despite only 7 % (*n* = 5) feeling this was the right time for this discussion [22]. Despite this, some midwives felt that women had decided where to give birth prior to PoB discussions occurring. This was thought to be for cultural reasons, such as family pressures, as well as the societal expectation that babies should be born in hospital. Other influences included parity, with midwives in the study by Kirkham et al [21] feeling that multiparous women knew what they wanted and were unlikely to change their minds following a discussion of PoB options.

Regarding informed choice, Kirkham et al [21] reported that some midwives felt it was up to individual women to find out their birth options, rather than being given all the information. Davis et al [16] described how midwives reported allowing women to consider their options by exploring their fears and expectations with them and building up their confidence about giving birth. This continued revisiting of PoB options with women was reported to often lead to women who had initially wanted an obstetric birth changing their minds.

#### Influence of midwifery colleagues

Two studies described how tensions between midwifery colleagues were felt to negatively impact on midwives’ PoB discussions with women. In Barber et al [12], midwives reported how they sometimes had difficulty engaging with their midwifery colleagues, due to differences in opinion around the promotion of home and MLU births. The RCM [18] study described how midwives reported a lack of promotion of homebirth amongst their midwifery colleagues and perceived how the unsupportive attitudes of some midwives towards homebirth meant that women were not always presented with adequate or appropriate information around where they could choose to give birth.

#### Review 2: Interventions to support midwives PoB discussions with women

Characteristics of the five studies which met the inclusion criteria are shown in Table 3 [11–13, 21, 22]. Two studies were single-sited [11, 13], taking place in the maternity units of two large NHS Hospital Trusts. The other three were multi-sited [12, 21] and encompassed both hospital and community based maternity settings. Midwives either provided case-loading care, were hospital or community based, or worked in both settings. All of the studies were UK based and were published between 2001 and 2015. Four studies provided details of the number of midwifery participants, ranging from 35 to 177 [11, 12, 21, 22]. Two studies had qualitative [12, 21], two quantitative [11] and one mixed methods [13] designs. After being assessed for quality using CASP tools, four studies were found to have a high risk of bias [11–13] and one an unclear risk of bias [21]. Though the study designs were appropriate to address the research aims, many provided unclear reporting of their recruitment strategies, lack of rigour in data analysis, a lack of precision when reporting the findings and a failure to acknowledge potential confounders. Concerns about the generalisability of the findings and the failure of some studies to acknowledge how their findings fitted alongside the wider evidence, also increased their risk of bias. The studies also failed to demonstrate any robust evidence for the effectiveness of their interventions in supporting midwives PoB discussions with women.

#### Qualitative studies

Of the two qualitative studies, the first by Kirkham et al [21], undertook ethnographic work and interviews with midwives and aimed to evaluate how midwives’ used Midwives’ Information and Resource Service (MIDIRS) Informed Choice Leaflets, following training in how to use them with women. The second study, by Barber et al [12], aimed to implement educational, marketing and change management initiatives on and around informed choice and PoB (including an evidenced based guideline on birthplace choices and a ‘Where to be born?’ leaflet for women) in order to educate midwives in their role in providing informed choice to women around PoB discussions.

#### Quantitative studies

The first quantitative study by Walton et al [11], involved evaluation of a pilot controlled study, which aimed to examine whether midwife training in, and use of a ‘Birthplace’ app (a web application accessible on personal computer, tablet and smartphone) influenced women’s actual PoB. A control group of women was retrospectively analysed to explore whether access to the app influenced actual PoB. The second quantitative study, by Barber et al [22], aimed to evaluate which educational, marketing and change management initiatives on and around informed choice and PoB- which had been implemented in their previous study [12] – had impacted on midwives’ practice and women’s decisions.

#### Mixed methods study

The mixed methods study by Rogers et al [13] addressed midwifery practice, using workshops with midwives. The workshops aimed to improve midwives’ knowledge and confidence about their PoB discussions with women. Other interventions included a ‘decision aid’ tool and changes to shift patterns to provide more senior support.

### Study findings

#### Effectiveness of Intervention

None of the five studies provided sufficient evidence of effectiveness of the interventions in practice. Kirkham et al [21] found no evidence that MIDIRS informed choice leaflets effectively increased the proportion of women who reported exercising informed choice. There were no significant differences in planned hospital birth between the intervention and comparator groups.

Rogers et al [13] found that women who had attended the Birthplace workshops and received the decision aid tool, reported being more likely to be offered a choice of PoB (93 % before, 97 % following intervention) and to have received sufficient information on which to base their choice (no data reported). Admissions to low risk MLUs reduced from 1203 in 2010 to 900 in 2014, though the authors report that this represents a 10 % increase of women giving birth in MLUs (no all-births denominator reported). Of all births occurring in low risk settings, the proportion in an alongside MLU increased from 71 % (*n* = 859) to 86 % (*n* = 778), homebirths decreased from 6 % (*n* = 71) to 5 % (*n* = 43) and births in free-standing MLUs decreased from 23 % (*n* = 273) to 9 % (*n* = 79) (note the reduction in frequency across all settings).

The pilot study by Walton et al [11] found that 45 % (*n* = 103) of women had decided a preferred PoB setting at their 12 week appointment. This increased to 88 % (*n* = 208) at 36 weeks, following the introduction of the app at 25 weeks. However, the lack of a comparator group of women who did not have access to the app, makes it difficult to conclude whether this change might have occurred anyway. A survey tool revealed that midwives liked using the app, finding it helpful in communicating and understanding evidence-based information.

Barber et al [22] reported that women experienced no difference in the amount of information they were offered around choice of PoB by midwives before and after their education, marketing and change management intervention package was implemented. The number of women giving birth in midwife-led settings increased by 1 % before and after the intervention (*n* = 2414 and 2640 respectively). There was no change in the proportion of births in a stand-alone MLU (8 %, *n* = 754 and 855 respectively), a 1 % increase in co-located birth centres (14–15 %, *n* = 1393 and 1529 respectively), and a 1 % decrease in home birth (3 to 2 %, *n* = 267 and 256 respectively). Note the overall birth rate increased by 5 % between the before and after intervention.

Barber et al [22] reported that despite initial scepticism, over time, midwives increasingly engaged with the project and tried to ensure more women received the Birthplace Choices leaflet. Most midwives self-reported that they had found the ‘Where to be born?’ leaflet and multi-professional guideline on PoB useful, with 9 % (*n* = 14) stating they had subsequently changed their practice [22]. However, Barber et al. [22] acknowledged that medical dominance, the prevailing culture of hospital birth being the norm, philosophical differences between and within professional groups and a tension for midwives between providing a service and accessing education, all limited the effectiveness of the intervention.

#### Influences on intervention implementation

Unlike the other studies in the review, Kirkham et al [21] explored some of the reasons why their intervention was not successfully implemented in practice. Kirkham et al [21] revealed that the MIDIRS informed choice leaflets were not always distributed appropriately. Some midwives failed to distribute the leaflets, feeling it contraindicated their personal views and philosophies on promoting births in low risk settings. Others were reported to place little value on the leaflets and so maintained their past practices. Some midwives were reported to use the leaflets inappropriately, either giving them to women at the wrong stage of pregnancy or using them to pre-empt discussion around PoB, as a substitute for conversation and to save time. Very few midwives discussed the content of the leaflets with women or distributed them according to need, as planned.

The study also found that some midwives felt women from non-English speaking backgrounds lacked access to informed choice through the MIDIRS leaflets as they were only produced in English, highlighting the inequalities around informed choice which exist [21]. In addition, some midwives assumed decision-making responsibility for women when deciding whether to distribute the leaflets, based on whether women would be able to read them, or should have access to the range of birth place options. This was done by stereotyping women according to their social background, age, literacy levels, or because midwives felt they should not be granted access to information which might sway their decision-making. In these situations midwives were reported to either omit certain PoB options during their discussions with women, or present them in such a way that women would be unlikely to disagree with their suggestions [21].

These influences impacting on the successful implementation of the intervention mirror the findings from review 1, which suggest that midwives often alter the content of their PoB discussions according to pre-formed assumptions about the needs of different women. Review 1 reports differing opinions of midwives in promoting birth in MLU and home settings, something which is confirmed in review 2, as Kirkham et al [21] describe how midwives personal views and philosophies around PoB often inhibited their engagement with the MIDIRS informed choice leaflets.

#### Limitations of this review

Every effort has been taken to provide a comprehensive and systematic literature review. However, some relevant studies may not have not been included, for example if they contained information on midwives views on PoB discussions with women, where this was not the main focus of the study. In addition, the review only included studies carried out in Europe, Australasia and North America, as it was felt unlikely that studies around PoB discussions in other continents would yield relevant findings, due to differing birthing contexts and environments. However, it is possible that some relevant studies might have been missed as a result. Some of the information required for the review was not documented in the study papers and despite contacting the authors, no response was received from one of the authors.

## Discussion

The review has identified that there is limited evidence regarding midwives’ perspectives of their discussions with women about where to give birth. The moderate to high risk of bias for most studies and the incomplete reporting of the study findings reduces their credibility. In addition the number of studies included for each type of PoB intervention was small, increasing the likelihood of chance findings. However, despite these methodological limitations, the findings offer some interesting insights. This review complements existing work, offering the first review which focuses specifically on how midwives carry out PoB discussions with women, from midwives perspectives, whereas most research has tended to focus on women’s perspectives. It has also highlighted that a paucity of interventions aimed at improving PoB discussions exist and that the studies reporting on this are of moderate to poor quality.

The findings suggest that numerous factors impact on midwives’ views of their discussions with women about their PoB options. Most commonly identified were organisational and professional norms meaning limited options being presented to women, with birth in an OU being expected. The promotion of birth in non-OU settings as ‘normal’, rather than ‘abnormal’ needs to be facilitated to change the current culture whereby women who choose non-OU births are often perceived to be risk-takers [23]. It is plausible that prior to the publication of the Birthplace study [1], a lack of robust evidence was available to support birth outside of an OU. The Birthplace study findings [1] have gone some way to highlighting the comparable safety of births in MLUs with births in OU settings for low risk, multiparous women, presenting a compelling argument for offering these women the full range of PoB options. However, seven of the studies included in the review were carried out prior to Birthplace [1] being published, suggesting that its findings have not yet increased the research undertaken and disseminated in this area. This needs addressing and more work needs to be done to translate this evidence into practice and change the cultural barriers which inhibit the promotion of alternative birth settings. In doing so, organisational and inter-professional tensions between midwives and their obstetric colleagues that have been shown to exist in this review, may abate to some extent, giving midwives more freedom to promote the full range of birth place options to women.

Another common theme identified in this review was the variation in the knowledge and confidence of midwives in discussing PoB options with women, especially with regard to homebirth. Strategies to improve midwives’ confidence levels could be facilitated by providing student midwives with more exposure to different birth settings throughout their training, as well as working to ensure that qualified midwives are given the opportunity to rotate through different practice settings. Increasing the level of insight and awareness that midwives have of alternative birth settings could also be achieved through regular managerial team meetings between hospital, community and homebirth teams, to highlight the complexities, practicalities and challenges that each setting has to offer.

The review has identified variation in how midwives talk to women about their options for where to give birth, due to differing perspectives on what they feel women need to know and what influences their decision-making processes. If PoB discussions are really going to promote choice of place of birth, the creation of a pragmatic condensed dialogue containing standard information is required, to support midwives in providing women with a baseline level of information about their PoB options that is relevant and comprehensible, enabling women to make an informed decision about where to give birth. The recently published NICE recommendations on PoB sets out guidance for midwives about what information they should be conveying to women about their options for where to give birth [4]. However, these NICE recommendations may be too lengthy and impractical for midwives, as they lack the time or capacity to translate the recommendations into their practice and convey them to women in a concise way. A more condensed, understandable and versatile dialogue needs to be produced, that can be implemented in the clinical setting and that can be incorporated into midwives’ routine antenatal appointments with women. Consideration of when the optimum time for this standardised PoB discussion might be is necessary, to ensure that women are ready to engage with this information, rather than automatically being given it at the booking visit, when they may be overloaded with information and not yet ready to consider their PoB options.

The review has identified a need for a more robust evaluation of any interventions designed to improve the quality of PoB discussions between midwives and women. The five interventions eligible for inclusion in the review were of a low to moderate quality and their findings should be interpreted with caution, due to the limited evidence for their effectiveness. When thinking about implementing any intervention into practice, robust evaluation should be encouraged and the evaluation of methods for getting evidence into practice is essential to informing good quality care [24]. None of the intervention studies in this review showed any evidence of using a structured approach to implementation, therefore it is unsurprising that none of the interventions were found to make a difference in practice. Systematic approaches to characterising and designing behaviour change interventions, such as the ‘COM-B’ system, developed by Michie et al [25] may be useful in improving the successful implementation of any interventions designed to improve midwives’ PoB discussions with women. The ‘COM-B’ is focused around understanding the three necessary conditions required to generate behaviour change: capability, opportunity and motivation [25]. Capability refers to the ability of the individual to psychologically and physically engage in the activity concerned, whilst opportunity is concerned with both social and environmental factors that make the behaviour more or less likely to succeed within a particular context. Motivation relates to both the automatic and reflective processes that guide our behaviour, such as emotional cues, reinforcement, incentivisation and goal-setting [25]. By selecting interventions which have been designed to account for these conditions, behaviour change is more likely to be achieved and maintained [25].

The interventions in review 2 demonstrated limited evidence of midwives agreeing to and being involved in the design, implementation and maintenance of the interventions, limiting their ability to facilitate changes to their clinical practice. This lack of co-production may have contributed to the lack of effectiveness of the interventions. Co-produced research aims to cross professional and organisational boundaries, so that the different groups involved actively participate in the production, interpretation and implementation of the findings [26, 27]. All of the interventions in this review involved midwives in delivering the intervention and engaged with them at certain stages of the study process, through for example, the formation of midwifery working groups [12] and focus groups with midwives [15]. However, co-production in its true sense was not achieved, as midwives were not consistently involved in agreeing on what changes were needed and what this change might look like. Engaging with midwives throughout the design, implementation and maintenance stages of this process is more likely to lead to sustainable and effective interventions being achieved in practice. This could be through listening to midwives’ views and perspectives on different birth settings, such as homebirth, and by responding to their thoughts on how different interventions can be used to promote the effective communication of PoB information to women.

## Conclusion

This review has identified the main influences impacting on midwives’ discussions with women about where to give birth and has highlighted that to date any interventions aimed at improving the way midwives carry out these PoB discussions with women have been lacking in effectiveness and have been of moderate to low quality. The review has highlighted the need for a condensed, pragmatic, understandable dialogue containing standard information about PoB options, to support midwives in providing women with a baseline level of information. It has also identified the need for a more robust and systematic evaluation of interventions designed to improve the quality of PoB discussions. By engaging with co-produced research, more effective interventions can be designed, implemented and sustained.

The review findings have identified a need for further high quality research to be undertaken to explore what the main influences on midwives’ PoB discussions with women are, in order to identify appropriate strategies and interventions to improve these discussions, so women can make fully-informed choices about their care. As such, this systematic review has been used to inform a larger, focus group study (to be published at a later date), exploring from midwives perspectives, how they discuss with women their options for where to give birth and any challenges and barriers to doing so. The findings from this study will be used to identify any priorities for change and possible solutions/interventions for changing the way that these discussions are undertaken in clinical midwifery practice, with the aim of moving midwives’ discussions with women about where to give birth forward and to promote choice for women where appropriate.

## Abbreviations

MLU: Midwifery-led unit
OU: Obstetric unit
PoB: Place of Birth
